# Supplementary material for: MetaRibo-Seq measures translation in microbiomes
Source: Nat Commun. 2020 Jun 29;11:3268. doi: 10.1038/s41467-020-17081-z (PMC7324362; doi:10.1038/s41467-020-17081-z)
Supplement: Supplementary file 10 — Supplementary Data 7 [file 41467_2020_17081_MOESM10_ESM.zip › File2/Confidence_VeryHigh_Taxonomy/426383_out.krona.html]

Javascript must be enabled to view this page.

members
magnitude
magnitudeUnassigned
count
unassigned
taxon
rank

426383\_out

26

26
superkingdom
2

26
phylum
1239


SRS024663\_contig\_number\_2614
species
1262991
1

186801
class
25

25
186802
order

1
family
186803

1
genus
572511

species
33039
1

SRS015782\_contig\_number\_35344

186806
family
18

18
1730
genus

species
142586
18

SRS015431\_contig\_number\_76330SRS018623\_contig\_number\_contig-100\_287.125788SRS019068\_contig\_number\_23325SRS050941\_contig\_number\_19471SRS050998\_contig\_number\_16061SRS052697\_contig\_number\_30400SRS058070\_contig\_number\_29593SRS075773\_contig\_number\_contig-100\_17.220759SRS1041136\_contig\_number\_19866SRS104693\_contig\_number\_12996SRS142923\_contig\_number\_contig-100\_6.120577SRS143148\_contig\_number\_24178SRS144135\_contig\_number\_32376SRS144362\_contig\_number\_43251SRS146812\_contig\_number\_63373SRS148159\_contig\_number\_46711SRS148784\_contig\_number\_contig-100\_236.161075SRS893383\_contig\_number\_contig-100\_211.171875

4
family
31979

4
1485
genus

1
2293030
species

SRS142503\_contig\_number\_17405


SRS014923\_contig\_number\_43964SRS050925\_contig\_number\_18062SRS051031\_contig\_number\_33506
59620
species
3

family
216572
2

2
459786
genus


SRS143780\_contig\_number\_20498
1
species
876091


SRS098881\_contig\_number\_1221
species
1945593
1
